# Excess risk of cancer in patients with primary venous thromboembolism: a national, population-based cohort study

**DOI:** 10.1038/sj.bjc.6601964

**Published:** 2004-06-29

**Authors:** J T Murchison, L Wylie, D L Stockton

**Affiliations:** 1 Department of Radiology, The Royal Infirmary of Edinburgh-Little France, 51 Little France Crescent, Old Dalkeith Road, Edinburgh EH16 4SA, UK; 2 Scottish Cancer Intelligence Unit, NHS Scotland, Information and Statistics Division, Trinity Park House, Edinburgh, EH5 3SQ, UK

**Keywords:** venous thromboembolism, record linkage, risk

## Abstract

We conducted a nationwide, retrospective cohort study assessing the risk of cancer in VTE patients diagnosed in Scotland in 1982–2000. Significantly elevated risks of cancer were sustained for 2 years after VTE diagnosis, most notably for ovarian tumours and lymphomas. Younger patients were at an increased relative risk from this association.

Trousseau (1872) observed the association between thrombotic phenomena and cancer as far back as 1865. Since then, numerous studies have documented the increased risk of cancer patients developing DVT and PE. More recently, association has been made between initial presentation with venous thromboembolism (VTE) and subsequent increased risk of cancer. This association has been investigated in several studies, but conclusions vary widely from no excess risk (O'Connor *et al*, 1984; Griffin *et al*, 1987) to a definite increased risk (Golgberg *et al*, 1987; Prandoni *et al*, 1992; Baron *et al*, 1998; Sorensen *et al*, 1998). Results of the time span of increased risk after DVT/PE diagnosis also vary, as do the types of cancer involved and the age groups that are at highest risk. This study was designed to determine (1) if there is an increased incidence of cancer in a large population-based cohort of Scottish patients with a new diagnosis of VTE, (2) to investigate any excess risk in relation to time since, and age at diagnosis of VTE and (3) to identify which cancers, in a Scottish population, are particularly associated with previous VTE.

## MATERIALS AND METHODS

### Patients

The Information and Statistics Division (ISD) of the National Health Service (NHS) in Scotland has linked (at time of analysis), using probability matching, information on all Scottish hospital inpatient discharges (1981–July 2002), death records (1981–2002) and cancer registry records (1981–2000). For this study, all records for patients diagnosed with a DVT (ICD9 451.1 and ICD10 I26, I80.1, I80.2), or PE (ICD9 415.1 and ICD10 I26) between 1982 and 2000 were selected from the linked database, giving a population-based database covering 19 years. Individuals presumed to be visitors who did not have a Scottish postcode and for whom follow-up data would not be available were excluded. To reduce the number of patients with secondary VTE, patients who had undergone surgery in the 6 weeks prior to VTE were excluded, as were pregnant females. Due to the difficulties in coding, we were unable to exclude those with haemostatic defects (thrombophilia). Follow-up for the occurrence of cancer was from the initial diagnosis of DVT/PE until the end of 2000, because the cancer records were not complete beyond this point. Hospital inpatient discharge data were available from 1981 onwards; however, the starting date was restricted to 1982 to reduce the erroneous inclusion of cases that had recurrent VTE.

Cancer diagnosis following DVT/PE was included if it was a first diagnosis of malignant cancer. Patients with a previous primary malignant cancer were excluded from the study. Only first cancers were included to avoid cancer itself being a confounding factor for a subsequent cancer, through genetic susceptibility, shared risk factors, or the effects of therapy. Data for non-melanoma skin cancer were excluded, as data are less likely to be complete for this common and usually nonfatal condition that often does not require hospital admission. Patients with cancer diagnosed within 1 month of VTE diagnosis were excluded.

### Statistical analysis

The expected number of cases of cancer in the cohort of VTE patients was calculated on the basis of Scottish national incidence rates of first malignant cancer according to age (5-year age bands to 85+), sex and period of diagnosis (1982–86, 1987–91, 1992–96, 1997–2000). Multiplying the number of person years of observation accumulated by the cohort (calculated from the date of diagnosis of VTE to the date of cancer diagnosis, date of death, or 31/12/2000, whichever came first) by the national incidence rates (by age, sex and time period) yielded the expected number of cancers in the VTE cohort if they were exposed to the same risk as the general population, calculating person-years for each subgroup (age at VTE diagnosis and time since VTE diagnosis) separately. The ratio of observed to expected cases gives the standardised incidence ratio (SIR) or relative risk, and confidence intervals were estimated assuming that the observed number of cases follows a Poisson distribution.

## RESULTS

In total, 77 572 patients were identified with DVT/PE or both diagnosed between 1981 and 2000. After applying the exclusion criteria, our cohort contained 59 534 patients, of whom 55% were female and 45% male. In all, 11.1% were ⩽39, 21.2% were 40–59, 22.4% 60–69, 26.7% 70–79 and 18.7% 80+. The median duration of follow-up was 32 months.

### Overall risk of developing cancer

Over the 19-year periods, 4441 (7.5%) patients were diagnosed with a first primary cancer after (at least 1 month after) their VTE diagnosis. The standardised incidence rate (SIR) for all cancers was 1.28 (CI 1.25–1.33) compared to that expected based on the incidence of first malignancies in Scotland.

### Risk of cancer in relation to time since, and age at diagnosis of VTE

For all malignancies combined, there was a high excess risk of being diagnosed with cancer (SIR 4.2, CI 3.9–4.5) within 1–6 months after diagnosis of VTE, with a slowly declining but still significant excess risk for each 6-month follow-up period up to 2 years. The risk was significantly raised for all individual malignancies calculated, but of particular note with SIRS greater than 5.0 were cancers of the ovaries, and Hodgkins and non-Hodgkins lymphoma (Table 1

**Table 1:** Observed and standardised incidence ratios (SIR) of first cancers diagnosed among patients with previous DVT/PE, in Scotland (1982–2000)

**Cancer group**	**ICD10 code**	**1 month to 1 year after VTE diagnosis**		**1st to 2nd year after VTE diagnosis**		**3rd to the 5th year after VTE diagnosis**	
Oesophagus	C15	21	1.4 (0.87–2.18)	28	2.1 (1.36–2.97)	29	1.0 (0.63–1.36)
Stomach	C16	77	3.2 (2.50–4.01)	25	1.1 (0.73–1.69)	45	0.9 (0.67–1.24)
Colon	C18	127	2.9 (2.43–3.48)	49	1.2 (0.90–1.62)	104	1.2 (0.94–1.40)
Rectum and anus	C19–C21	28	1.3 (0.84–1.84)	18	0.9 (0.53–1.41)	42	0.9 (0.67–1.25)
Liver	C22	17	3.9 (2.26–6.23)	2	0.5 (0.04–1.82)	12	1.3 (0.67–2.32)
Gallbladder and biliary tree	C24	5	2.6 (0.80–6.03)	3	1.7 (0.30–4.96)	4	1.0 (0.26–2.57)
Pancreas	C25	55	4.1 (3.07–5.32)	16	1.3 (0.74–2.12)	23	0.8 (0.53–1.27)
Lung	C33–C34	305	3.0 (2.71–3.39)	125	1.4 (1.13–1.62)	183	0.9 (0.77–1.03)
Breast	C50	70	1.6 (1.20–1.96)	54	1.3 (0.98–1.71)	98	1.1 (0.87–1.30)
Corpus	C54	15	2.7 (1.46–4.38)	3	0.6 (0.10–1.72)	10	0.9 (0.42–1.63)
Cervix	C53	15	3.6 (2.01–6.02)	5	1.3 (0.41–3.13)	12	1.5 (0.74–2.54)
Ovary	C56	64	7.1 (5.48–9.03)	11	1.3 (0.66–2.40)	22	1.2 (0.76–1.84)
Prostate	C61	112	3.0 (2.42–3.54)	41	1.1 (0.81–1.54)	111	1.3 (1.08–1.58)
Bladder	C67	50	2.0 (1.51–2.67)	16	0.7 (0.40–1.15)	59	1.2 (0.88–1.49)
Kidney and urothelium	C64, C68	37	4.3 (3.00–5.86)	9	1.1 (0.51–2.14)	22	1.2 (0.76–1.85)
Meninges and brain	C70–C72	12	2.5 (1.28–4.40)	5	1.1 (0.35–2.66)	9	0.9 (0.41–1.75)
Non-Hodgkins lymphoma	C82–C85	64	5.1 (3.95–6.51)	19	1.6 (0.98–2.56)	24	0.9 (0.58–1.37)
Hodgkins disease	C81	7	6.0 (2.34–12.4)	2	1.9 (0.17–6.83)	2	0.9 (0.08–3.17)
Multiple myeloma	C88, C90	24	3.8 (2.42–5.72)	10	1.7 (0.82–3.19)	15	1.2 (0.63–1.90)
Leukaemia	C91–C96	20	1.9 (1.14–2.93)	10	1.0 (0.48–1.90)	20	0.9 (0.55–1.41)
Other cancers^a^		220	3.2 (2.82–3.70)	74	1.2 (0.92–1.48)	174	1.2 (1.04–1.42)
All cancers^a^	C00–C96	1345	2.9 (2.74–3.06)	525	1.2 (1.12–1.33)	1020	1.1 (0.99–1.13)

a Excluding nonmelanoma skin cancer (ICD10: C44).

). The risk after 2 years was very similar to that expected in the general population. This excess risk within the first 2 years was seen in all age groups, but declined as age increased (Table 2

**Table 2:** Standardised incidence ratios (SIR) with 95% confidence intervals of first cancers diagnosed among patients with previous VTE assessed according to age band and time interval after diagnosis of VTE

	**Age at VTE diagnosis**				
**Time since VTE diagnosis**	**20–39**	**40–59**	**60–79**	**80+**	**All ages**
1–6 months	5.8 (2.7–11)	7.5 (6.4–8.8)	4.2 (3.8–4.5)	3.0 (2.6–3.5)	4.2 (3.9–4.5)
6–12 months	2.3 (0.6–5.8)	2.3 (1.8–3.0)	1.7 (1.5–1.9)	1.5 (1.2–1.9)	1.7 (1.5–1.9)
12–18 months	1.0 (0.1–3.7)	1.5 (1.0–2.1)	1.3 (1.1–1.5)	1.0 (0.8–1.3)	1.2 (1.1–1.4)
18–24 months	2.2 (0.6–5.6)	1.6 (1.1–2.2)	1.1 (0.9–1.3)	1.4 (1.1–1.8)	1.2 (1.1–1.4)
24–30 months	1.5 (0.3–4.5)	1.1 (0.7–1.6)	1.0 (0.8–1.2)	1.1 (0.8–1.4)	1.0 (0.9–1.2)
30–36 months	0.5 (0.0–3.1)	0.8 (0.5–1.3)	1.1 (1.0–1.4)	1.0 (0.7–1.5)	1.1 (0.9–1.3)
36–48 months	1.1 (0.3–2.7)	1.2 (0.9–1.5)	1.1 (1.0–1.3)	1.1 (0.8–1.4)	1.1 (1.0–1.2)
48–60 months	1.6 (0.6–3.5)	1.2 (0.9–1.5)	1.0 (0.9–1.2)	1.0 (0.7–1.3)	1.1 (0.9–1.2)
5–10 years	1.2 (0.7–1.8)	1.1 (1.0–1.3)	1.0 (0.9–1.1)	0.9 (0.8–1.2)	1.0 (1.0–1.1)

), with excess risk approximately twice as large in patients aged under 60 at VTE diagnosis compared to those aged 60 or over (Table 2).

## DISCUSSION

**Figure 1:**
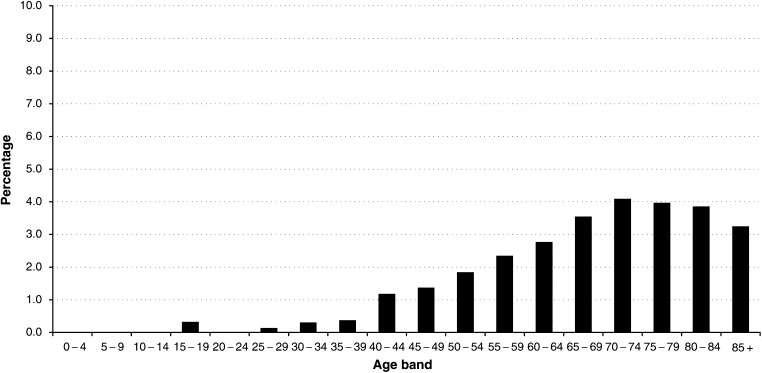
Percentage of patients who developed cancer within 1–12 months after the first episode of VTE in relation to the total number of VTE patients, by age at VTE diagnosis.

We evaluated the association between VTE and subsequent diagnosis of cancer in a large cohort, and demonstrated a definite increased risk of being diagnosed with malignancy after a primary episode of VTE. The risk is particularly marked within 1–12 months after VTE diagnosis. Some studies have suggested that a small increase in risk persists many years after the diagnosis of VTE (Golgberg *et al*, 1987; Prandoni *et al*, 1992). Our overall figures show an excess risk sustained for the first 2 years after VTE diagnosis, and thereafter, the risk returns to that expected based on background rates. In our cohort, the excess risk of cancer was highest in patients aged under 60 at VTE diagnosis; however, as malignancy has an increased prevalence with increasing age, older patients have a greater absolute risk of malignancy after a first episode of VTE, with one in 25 60–75-year olds developing cancer within 1 year (Figure 1

). The results of this study can only be generalised to patients admitted to hospital, and relies on accurate discharge coding, cancer registration and linkage. As almost all acute illnesses are treated in NHS hospitals in Scotland and as over the period of the study the practice was to admit acute VTE to hospital for stabilisation of anticoagulation, it is likely that most diagnosed cases have been included. There may, however, be other undiagnosed cases that we are unaware of, as VTE is a diagnosis that is difficult to make clinically and which is often overlooked. The accuracy of discharge coding data in Scotland is estimated at around 90% (Harley and Jones, 1996) and the quality of cancer registry data is also high (Brewster *et al*, 1994, 1997). Mismatched records in the Scottish record linkage system occur in less than 2% of cases (Kendrick and Clarke, 1993). Any bias introduced by miscoding or mismatched records is likely to underestimate the risk. Bias occurring due to loss of subjects through migration is likely to be small and would also lead to an underestimation of risk.

Although our analysis had the strength of being a large population-based study, our data lacked clinical detail, in particular about risk factors for thromboembolism and the stage of the cancer at diagnosis.

This study provides further evidence for the increased risk of developing cancer after an episode of primary VTE, and shows that this persists for a 2-year period; however, it does not provide an answer to the question whether such patients should be screened for occult malignancy. It is reported that, in cases where cancer is diagnosed after an episode of VTE, the cancer is often advanced and the outcome is very poor, with a 1-year survival of only 12% (Sorensen *et al*, 2000). It is also uncertain whether earlier diagnosis changes outcome, and any perceived benefits of earlier diagnosis must be weighed against the psychological and physical morbidity and discomfort associated with extensive investigations (Nordstrom *et al*, 1994; Prins *et al*, 1994). Simple clinical and diagnostic methods of screening, in patients with idiopathic DVT/PE, seem sensible and are recommended in other studies (Monreal *et al*, 1991; Piccioli *et al*, 1996; Sorensen *et al*, 2000).
